# Oncological outcomes of laparoscopic versus open nephroureterectomy for the treatment of upper tract urothelial carcinoma: an updated meta-analysis

**DOI:** 10.1186/s12957-021-02236-z

**Published:** 2021-04-21

**Authors:** Radosław Piszczek, Łukasz Nowak, Wojciech Krajewski, Joanna Chorbińska, Sławomir Poletajew, Marco Moschini, Krzysztof Kaliszewski, Romuald Zdrojowy

**Affiliations:** 1 Department of Urology and Oncologic Urology, Lower Silesian Specialist Hospital, Fieldorfa 2 Street, 50-556 Wroclaw, Poland; 2 Department of Urology and Urological Oncology, Wroclaw Medical University, Borowska 213 Street, 50-556 Wroclaw, Poland; 3 Centre of Postgraduate Medical Education, Marymonecka 99/813 Street, 01-813 Warsaw, Poland; 4 Klinik für Urologie, Luzerner Kantonsspital, Spitalstrasse, 6004, 16 Lucerne, Switzerland; 5 Department of General, Minimally Invasive and Endocrine Surgery, Wroclaw Medical University, Borowska 213 Street, 50-556 Wroclaw, Poland

**Keywords:** Upper tract urothelial carcinoma, Open nephroureterectomy, Laparoscopic nephroureterectomy

## Abstract

**Background:**

During the past two decades, laparoscopic radical nephroureterectomy (LRNU) has been proposed as an alternative technique to open radical nephroureterectomy (ORNU) and has become increasingly accepted for the treatment of patients with upper tract urothelial carcinoma (UTUC). Nevertheless, the oncologic efficacy of LRNU remains controversial, especially for the treatment of locally advanced (T3/T4 and/or N+) UTUC. In this meta-analysis, we aimed to cumulatively compare the oncological outcomes of LRNU versus ORNU.

**Materials and methods:**

The present meta-analysis was performed according to the Preferred Reporting Items for Systematic Reviews and Meta-Analyses (PRISMA) statement. A search was conducted of three electronic databases, namely, Medline, Embase, and Cochrane Library. Outcome measurements of cancer-specific survival (CSS), overall survival (OS), intravesical recurrence-free survival (IVRFS), and recurrence-free survival (RFS), including hazard ratios (HRs) and 95% confidence intervals (CIs), were extracted and pooled.

**Results:**

Eighteen articles published from 2007 to 2020 were included in the final quantitative analysis. One study was a randomized controlled trial (RCT), and the remaining articles had a retrospective design. Among a total of 10,730 participants in the selected papers, 5959 (55.5%) and 4771 (44.5%) underwent ORNU and LRNU, respectively. The results of pooled analyses revealed no significant differences in CSS (HR 0.84, 95% CI 0.60–1.19, *p* = 0.33), OS (HR 0.84, 95% CI 0.62–1.13, *p* = 0.25), IVRFS (HR 1.08, 95% CI 0.85–1.39, *p* = 0.52), and RFS (HR 1.09, 95% CI 0.94–1.25, *p* = 0.26) between LRNU and ORNU groups. Furthermore, the results of subgroup analyses for pT3/T4 and pTany N+ populations did not confirm any statistically significant differences between LRNU and ORNU in terms of any survival parameter.

**Conclusions:**

Our present meta-analysis of current evidence suggests that LRNU and ORNU have comparable oncological outcomes in patients with UTUC, even in those with locally advanced disease. Further multicenter RCTs with large sample sizes and uniform data regarding specific surgical procedures, such as bladder cuff excision, are required to establish definitive conclusions.

**Supplementary Information:**

The online version contains supplementary material available at 10.1186/s12957-021-02236-z.

## Introduction

Upper tract urothelial carcinoma (UTUC) is an uncommon neoplasm accounting for approximately 5–10% of all urothelial cancers [1]. It refers to any malignancies that arise from the urothelial lining of the upper urinary tract, from the calyceal system up to the ureteral opening into the bladder [1]. Although overall incidence of UTUC has decreased, the incidence of metastatic UTUC has increased in the recent years [2].

According to the European Association of Urology (EAU) guidelines, open radical nephroureterectomy (ORNU) with bladder cuff excision is the standard treatment for high-risk UTUC [3]. However, during the past two decades, laparoscopic radical nephroureterectomy (LRNU) has been proposed as an alternative technique to the open approach and has become increasingly accepted for UTUC treatment [4]. Nevertheless, a sole randomized controlled trial (RCT) raised the hypothesis that patients with UTUC might have worse oncological outcomes if treated with a laparoscopic approach, particularly for locally advanced cases [5]. Since the publication of the last systematic review and meta-analysis [6], several studies that compared the oncological outcomes between LRNU and ORNU have been published, including large multicenter trials with matched cohorts. Therefore, we sought to perform an updated quantitative synthesis of data from the available literature. In this meta-analysis, we aimed to compare the oncological outcomes of LRNU versus ORNU in patients with UTUC.

## Material and methods

### Search strategy

This meta-analysis was performed according to the Preferred Reporting Items for Systematic Reviews and Meta-Analyses (PRISMA) statement and the Cochrane Handbook for Systematic Reviews of Interventions [7, 8]. Study protocol was registered with PROSPERO (CRD42021239989). Two review authors (RP and LN) independently conducted a systematic search of three electronic databases, namely, Medline, Embase, and Cochrane Library. The most recent search was performed on 18 March 2021. Screening of the literature was conducted using the following terms/keywords: (“upper tract urothelial carcinoma” OR “upper urinary tract carcinoma” OR “upper tract” OR “UTUC” OR “UUTC”) AND (“laparoscopic” OR “laparoscop*” OR “LNU” OR “LRNU”) AND (“open” OR “conventional” OR “ONU” OR “ORNU” OR “surgery”). No specific time or language restrictions were applied. A cross-referenced search was also performed from articles selected for full-text review. Additional articles were screened from ahead of print articles in various urological journals.

### Selection criteria

We evaluated studies for inclusion and exclusion based on a predefined PICOS approach where the population (P), intervention (I), comparator group (C), outcome (O), and study design (S) were considered. The inclusion criteria were as follows: (P) studies that involved patients with UTUC; (I) patients who underwent LRNU; (C) compared with those who underwent ORNU; (O) cancer-specific survival (CSS), overall survival (OS), intravesical recurrence-free survival (IVRFS), and recurrence-free survival (RFS); and (S) prospective and retrospective trials. Prospective RCTs were considered eligible without any additional limitations, whereas retrospective papers had to provide data from multivariable analyses adjusted for at least one major confounder. Only studies reporting median follow up of a minimum of 12 months were included. To avoid small sample bias, only studies with a minimum of 50 patients were considered eligible for pooling survival estimates. Case reports, case series, conference abstracts, reviews, and letter to editors were excluded after the initial screening.

### Data extraction

After removal of duplicates, two review authors (RP and LN) independently screened titles and abstracts of the retrieved records using a standardized item form. All potentially eligible studies were evaluated as full text if available. In the case of multiple reports of the same cohort, the most complete data aggregated with the longest follow-up duration was selected. In the case of any discrepancy over inclusion of a particular study, a final decision was established after reaching a consensus with the other authors.

The following data were initially extracted: first author, year of publication, study region, study design, number of patients who underwent ORNU and LRNU, and median follow-up. Furthermore, the following clinicopathological data were retrieved: LRNU approach, method of bladder cuff excision, pathological tumor stage and grade, rates of patients with pathologically confirmed lymph nodes (LN) metastases, rates of patients with concomitant carcinoma in situ (CIS), rates of patients with lymphovascular invasion (LVI), rates of patients with positive surgical margins (PSM), and proportion of patients receiving neoadjuvant (NAC) and adjuvant chemotherapy (AC). Subsequently, the outcome measurements of CSS, OS, IVRFS, and RFS (including hazard ratios [HRs] and 95% confidence intervals [95% CIs]) were extracted. Missing information or clarifications were sought by contacting the primary authors; however, no additional data were received.

### Quality and risk of bias assessment

The quality of the selected studies was assessed independently by two review authors (WK and RP). The evaluation of the methodological quality of the non-RCT and RCT was performed according to the Newcastle–Ottawa Scale (NOS) [9] and Jadad Scale (JS) [10], respectively.

The risk of bias (RoB) was determined using the pragmatic approach for the evaluation of nonrandomized studies by examining the adjustments for confounders according to the Cochrane Handbook for Systematic Reviews of Interventions [8]. The articles were therefore reviewed based on the adjustment for the effects of the following confounders: age, pathological tumor stage, pathological tumor grade, concomitant CIS, presence of LN metastases, presence of LVI, AC administration, and PSM. The RoB of each study was assessed independently by two authors (RP and WK), and all disagreements were resolved by consultation with the other authors.

Finally, we assessed the potential publication bias. Because visual interpretation of the funnel plot asymmetry is inherently subjective and should be interpreted carefully due to several possible explanations, publication bias assessment was mainly based on the Egger’s and Begg’s asymmetry tests results.

### Statistical analysis

The statistical analysis was conducted using Review Manager 5.3 (The Nordic Cochrane Center, The Cochrane Collaboration, Copenhagen, Denmark) and Statistica 13.0 (TIBCO). From retrospective studies, we collected only reported multivariable HRs and 95% CIs without performing any effect summary estimation methods. The statistical significance of the pooled HRs was evaluated by the *Z* test. Statistical pooling of effect measures was based on the level of heterogeneity among studies. Significant heterogeneity was indicated by either ratio of > 50% in *I*^2^ statistics or *p* value of < 0.10 in Cochrane’s *Q* test, which led to the use of the random-effect model. When no significant heterogeneity was observed, fixed-effect models were used for calculations. Additionally, meta-regression and subgroup analyses were conducted in order to relate specific study-level variables to the statistical heterogeneity between the results of the studies. For all tests (other than Cochrane’s *Q* test), *p* < 0.05 was considered a statistically significant difference.

## Results

### Study population and risk of bias assessment

**Fig. 1:**
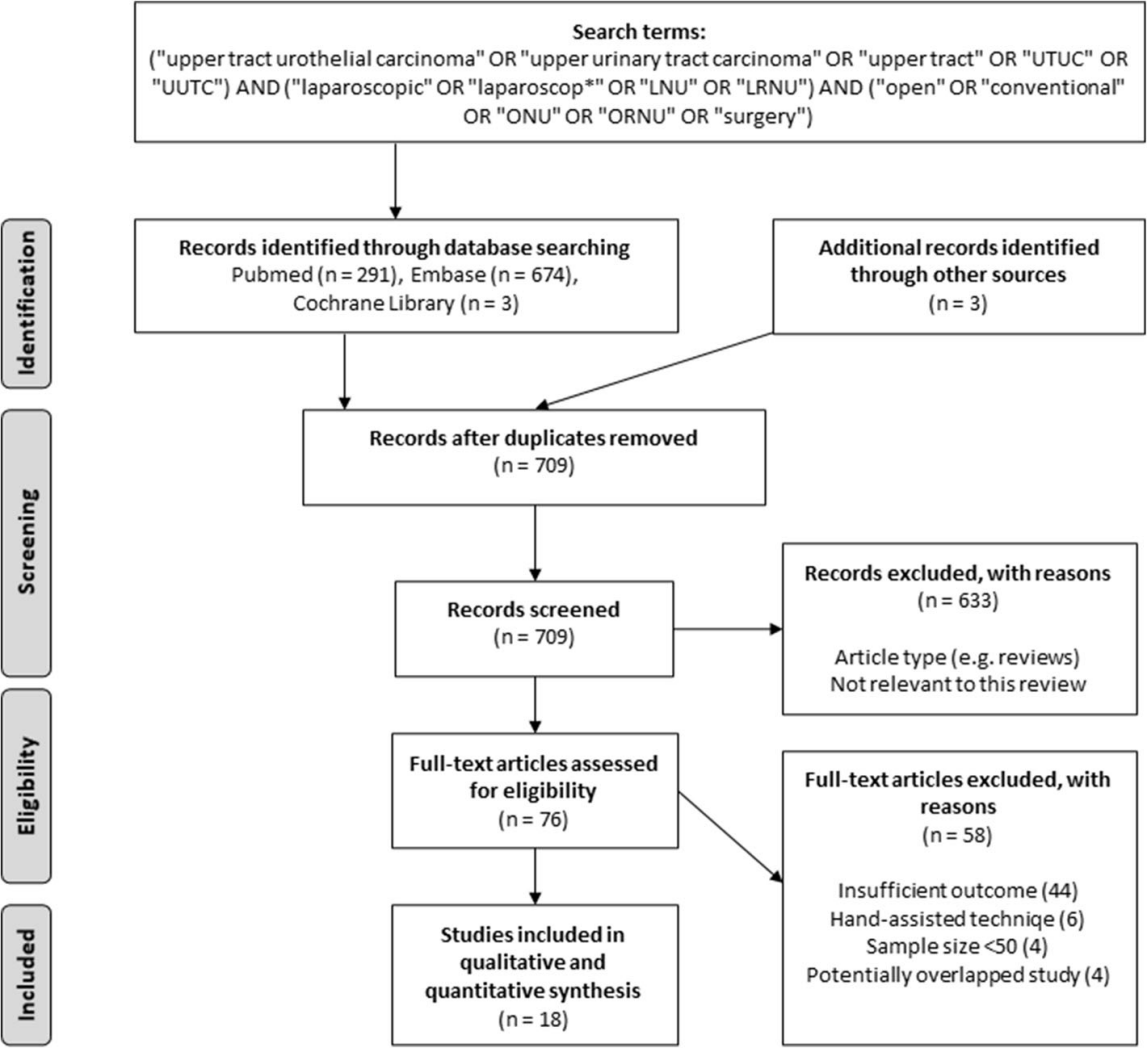
Flow diagram of study selection

The flow diagram of study selection with subsequent exclusions is presented in Fig. 1. Our search strategy initially identified 971 articles (968 from online databases and 3 from additional sources). Following deduplication (*n* = 709) and screening of the titles and abstracts, 633 studies were excluded because of inappropriate type (reviews, editorials, case reports, letters), irrelevance to present topic, or reporting robotic technique as an only comparative arm. Subsequently, 76 full-text articles were assessed for eligibility and 58 were ultimately excluded due to the following: insufficient outcome (*n* = 44), inclusion of hand-assisted laparoscopic technique as an only comparative arm (*n* = 6), small sample size (*n* = 4), and overlap with previously reported studies (*n* = 4). Thus, 18 articles published from 2007 to 2020 were included in the final meta-analysis [5, 11–27].

Table 1 summarizes the main characteristics of studies included in this meta-analysis. One study was an RCT [5], and the remaining articles had a retrospective design [11–27]. Among a total of 10,730 patients in the selected papers, 5959 (55.5%) and 4771 (44.5%) underwent ORNU and LRNU, respectively. The reported median follow-up (for whole or individual LRNU/ORNU cohorts) was longer than 2 years in the majority of the selected papers. Assessment of quality scores by the NOS showed that the scores of included retrospective studies ranged from 6 to 8, which were considered appropriate for this meta-analysis. Jadad Scale score for single included RCT was 4 (representing good methodological quality).

**Table 1:** Baseline characteristics of included studies

Study (year)	Country	Design	Duration	Number of patients (ORNU/LRNU)	Follow up, median (months) (ORNU/LRNU)	Extracted outcomes	NOS^a^/JS^b^
**Ariane** et al. (2012) [11]	France	R, *multi-institutional*	1995–2010	459/150	Whole cohort: 27	CSS, RFS	6^a^
**Azawi** et al. (2020) [12]	Denmark	R, *population-based registry*	2004–2017	321/1063	Whole cohort: 54	OS, IVRFS	6^a^
**Capitanio** et al. (2009) [13]	International	R, *multi-institutional*	1987–2007	979/270	Whole cohort: 49	CSS, RFS	8^a^
**Fairey** et al. (2013) [14]	Canada	R, *multi-institutional*	1994–2009	403/446	Whole cohort: 26.4	CSS, OS, RFS	7^a^
**Favaretto** et al. (2010) [15]	United States	R, *single-center*	2002–2008	109/53	Whole cohort: 23	IVRFS, RFS	6^a^
**Fradet** et al. (2014) [16]	Canada	R, *multi-institutional*	1990–2010	267/345	Whole cohort: 40.4	IVRFS	7^a^
**Kido** et al. (2018) [17]	Japan	R, *multi-institutional*	1995–2017	351/75*	41/35	CSS, OS, IVRFS, RFS	7^a^
**Kim HS** et al. (2016) [18]	Korea	R, *single-center*	1992–2012	271/100	57.6/38.8	CSS, OS	8^a^
**Kim SH** et al. (2019) [19]	Korea	R, *multi-institutional*	2000–2012	638/638**	37.8/44.3	CSS, OS, IVRFS	8^a^
**Kitamura** et al. (2014) [20]	Japan	R, *multi-institutional*	1995–2010	34/65	Whole cohort: 60	IVRFS	6^a^
**Koda** et al. (2007) [21]	Japan	R, *single-center*	1995–2005	27/79	Mean: 46.2/16.4	IVRFS	8^a^
**Lee** et al. (2019) [22]	Korea	R, *single-center*	2004–2017	161/137	Mean: 41.7/38.1	CSS, OS, IVRFS	8^a^
**Lenis** et al. (2018) [23]	United States	R, *population-based registry*	2000–2013	338/380	NR	OS	6^a^
**Moschini** et al. (2020) [24]	International	R, *multi-institutional*	2006–2018	757/757**	Whole cohort: 62	CSS, OS, RFS	8^a^
**Shigeta** et al. (2019) [25]	Japan	R, *multi-institutional*	1990–2015	72/72**	Whole cohort: 65.4	CSS, IVRFS	7^a^
**Simone** et al. (2009) [5]	Italy	RCT, *single-center*	2003–2006	40/40	Whole cohort: 41	CSS	4^b^
**Taweemonkongsap** et al. (2008) [26]	Thailand	R, *single-center*	2001–2007	29/31	Mean: 27.9/26.4	RFS	7^a^
**Walton** et al. (2011) [27]	International	R, *multi-institutional*	1987–2008	703/70	36/17	CSS, RFS	7^a^

*Inverse-probability weighted analysis

**Propensity-score matched analysis

*Abbreviations*: CSS, cancer-specific survival; IVRFS, intravesical recurrence-free survival; JS, Jadad Scale; LRNU, laparoscopic radical nephroureterectomy; NOS, Newcastle-Ottawa Scale; OS, overall survival; ORNU, open radical nephroureterectomy; R, retrospective; RCT, randomized controlled trial; RFS, recurrence-free survival

The detailed data regarding surgical techniques and clinicopathological characteristics of patients in selected articles are presented in Table 2. LRNU was performed using a transperitoneal and retroperitoneal route in six [5, 11, 18, 19, 22, 27] and three [17, 21, 26] studies, respectively. Transperitoneal or retroperitoneal access was reported in three publications [15, 20, 25], and the remaining six articles did not report the route that was used [12–14, 16, 23, 24]. The approach for bladder cuff excision in the LRNU groups was not specified or was heterogeneous in 11 papers [11–17, 20, 22–24, 27]. The distal ureter was managed uniformly via an open extravesical and laparoscopic extravesical approach in six [17–19, 21, 25, 26] and one [5] studies, respectively. In the vast majority of studies, regional LN dissection was performed selectively in cases in which LN involvement was suspected on preoperative imaging or was identified during surgery. In two papers, LN dissection was not performed in any case [5, 21]. Most studies did not report significant differences in pathological stage, grade, rates of concomitant CIS, LVI, and PSM, between the ORNU and LRNU groups. Eight studies [5, 12, 15, 17–19, 25, 26], reported similar rates of LN metastases between the ORNU and LRNU groups, and in eight studies [11, 13, 14, 16, 22–24, 27], rates of LN metastases were significantly higher in the ORNU group. Data regarding use of NAC were presented only in two selected papers [17, 23], whereas in other articles, patients receiving NAC were initially excluded or no data were available. Detailed data regarding AC administration were provided in 12 of 18 articles [14, 16–19, 21, 23–27].

**Table 2:** Surgical and clinicopathological characteristics of included studies

Study (year)	LRNU route	Management of distal ureter ^#^	Lymphadenectomy (%) (ORNU/LRNU)	Pathological stage ≥ pT3 (%) (ORNU/LRNU)	Pathological grade (%) (ORNU/LRNU)	Positive nodal status (%) (ORNU/LRNU)	Concomitant CIS (%) (ORNU/LRNU)	Present LVI (%) (ORNU/LRNU)	Positive surgical margins (%) (ORNU/LRNU)	NAC (%) (ORNU/LRNU)	AC (%) (ORNU/LRNU)
**Ariane** et al. (2012) [11]	TP	Mixed	Regional Selectively performed	pT3: 33.3/35.3 pT4: 6.3/1.3	G1: 8.5/7.3 G2: 36.2/27.3 G3: 55.3/65.3	10.2/4.7*	NR	19.2/18.7	NR	Excluded	NR
**Azawi** et al. (2020) [12]	NR	NR	Extent not described Whole cohort: 5%	pT3: 14.6/11.9 pT4: 5.6/1.7	Low grade: 46.4/24.8* High grade: 53.6/75.2	Whole cohort: 1.4%	NR	NR	NR	NR	NR
**Capitanio** et al. (2009) [13]	NR	Mixed No bladder cuff	Regional 42.4/24.4*	pT3: 31.3/21.9* pT4: 3.9/1.5	Low grade: 40.3/38.1 High grade: 59.7/61.9	7.2/2.2*	25.9/24.1	21.3/14.8*	NR	Excluded	Excluded
**Fairey** et al. (2013) [14]	NR	Mixed	Regional 34/25*	pT3: 25/26 pT4: 6/5	Low grade: 31/36 High grade: 69/64	10/4*	10/25	NR	11/10	NR	9/13
**Favaretto** et al. (2010) [15]	TP, RP	Mixed	Mainly regional 81/70	pT3: 32/32	Low grade: 12/9 High grade: 86/87	16/13	29/28	NR	NR	NR	NR
**Fradet** et al. (2014) [16]	NR	Mixed	Extent not described Selectively performed	NR	G1: 29.2/37 G2–G3: 70.8/63.0	11.2/4.3*	NR	NR	NR	Excluded	7.2/12.5*
**Kido** et al. (2018) [17]	RP	Open extravesical	Regional Selectively performed	pT3: 40/41 pT4: 4/0	Low grade: 6/1 High grade: 94/99	7.7/4	NR	39/23	4/0*	20/41*	3.3/0
**Kim SH** et al.(2016) [18]	TP	Open extravesical	Regional Selectively performed	pT3/T4: 41.3/34	Low grade: 30/35.1 High grade: 70/64.9	3.7/0	13.7/13	19.2/19	4.1/3	Excluded	24/20
**Kim HS** et al. (2019) [19]	TP	Open extravesical	Regional 44.5/45.6	pT3: 40.1/38.2 pT4: 1.4/1.1	Low grade: 27.9/31.8 High grade: 69.8/65.5	4.6/4.9	15.7/14.1	20.5/19.9	NR	Excluded	20.1/20.4
**Kitamura** et al. (2014) [20]	TP, RP	Mixed	Extent not described Selectively performed	pT3: 47/43	G1: 3/3 G2: 41/50 G3: 56/47.3	NR	NR	38/22	NR	NR	NR
**Koda** et al. (2007) [21]	RP	Open extravesical	Not performed	pT3: 25.9/35.4 pT4: 0/3.8	G1: 11.1/12.7 G2: 59.3/41.8 G3: 29.6/45.6	NR	NR	NR	NR	NR	14.8/12.7
**Lee** et al. (2019) [22]	TP	Mixed	Extent not described Selectively performed	pT3: 39.1/33.6 pT4: 10.6/1.5	G1: 0.6/0.7 G2: 42.2/54 G3: 57.1/45.3	36.6/19.7*	NR	41.6/25.5*	NR	NR	NR
**Lenis** et al. (2018) [23]	NR	NR	Extent not described 35.1/27.4	pT3: 35.9/34.9 pT4: 7/4.1	Low grade: 23.4/25.6 High grade: 76.6/74.4	12/6.1*	NR	NR	12.7/7.4*	3.2/1.2	15/12.1
**Moschini** et al. (2020) [24]	NR	NR	Regional Selectively performed	pT3–pT4: 35/34	Low grade: 53/60* High grade: 47/40	15/6*	21/18	32/27	NR	NR	16/14
**Shigeta** et al. (2019) [25]	TP, RP	Open extravesical	Regional Selectively performed	pT3: 100/100	Low grade: 31.9/41.7 High grade: 68.1/58.3	0/0	NR	56.9/48.6	NR	Excluded	38.9/29.2
**Simone** et al. (2009) [5]	TP	Laparoscopic extravesical	Not performed	pT3: 32.5/30	Low grade: 37.5/42.5 High grade: 62.5/57.5	0/0	NR	NR	NR	NR	In patients with metastases
**Taweemonkongsap** et al. (2008) [26]	RP	Open extravesical	Extent not described 31/64.5	pT3: 13.8/12.9 pT4: 0/3.2	Low grade: 34.5/58.1 High grade: 65.5/41.9	10.3/3.2	NR	NR	NR	NR	10.3/3.2
**Walton** et al. (2011) [27]	TP	NR	Regional 23.3/30	pT3: 27.9/27.1 pT4: 5.7/5.7	G1: 12.4/15.7* G2: 31.2/7.1 G3: 56.3/77.1	6.8/2.9*	11.1/17.1	18.8/22.9	NR	Excluded	7.7/17.1*

*Statistically significant difference

^#^Mixed—open and/or laparoscopic extravesical and/or endoscopic

*Abbreviations*: AC, adjuvant chemotherapy; CIS, carcinoma in situ; LRNU, laparoscopic nephroureterectomy; LVI, lymphovascular invasion; NAC, neoadjuvant chemotherapy; NR, not reported; ORNU, open nephroureterectomy; RP, retroperitoneal; TP, transperitoneal

**Fig. 2:**
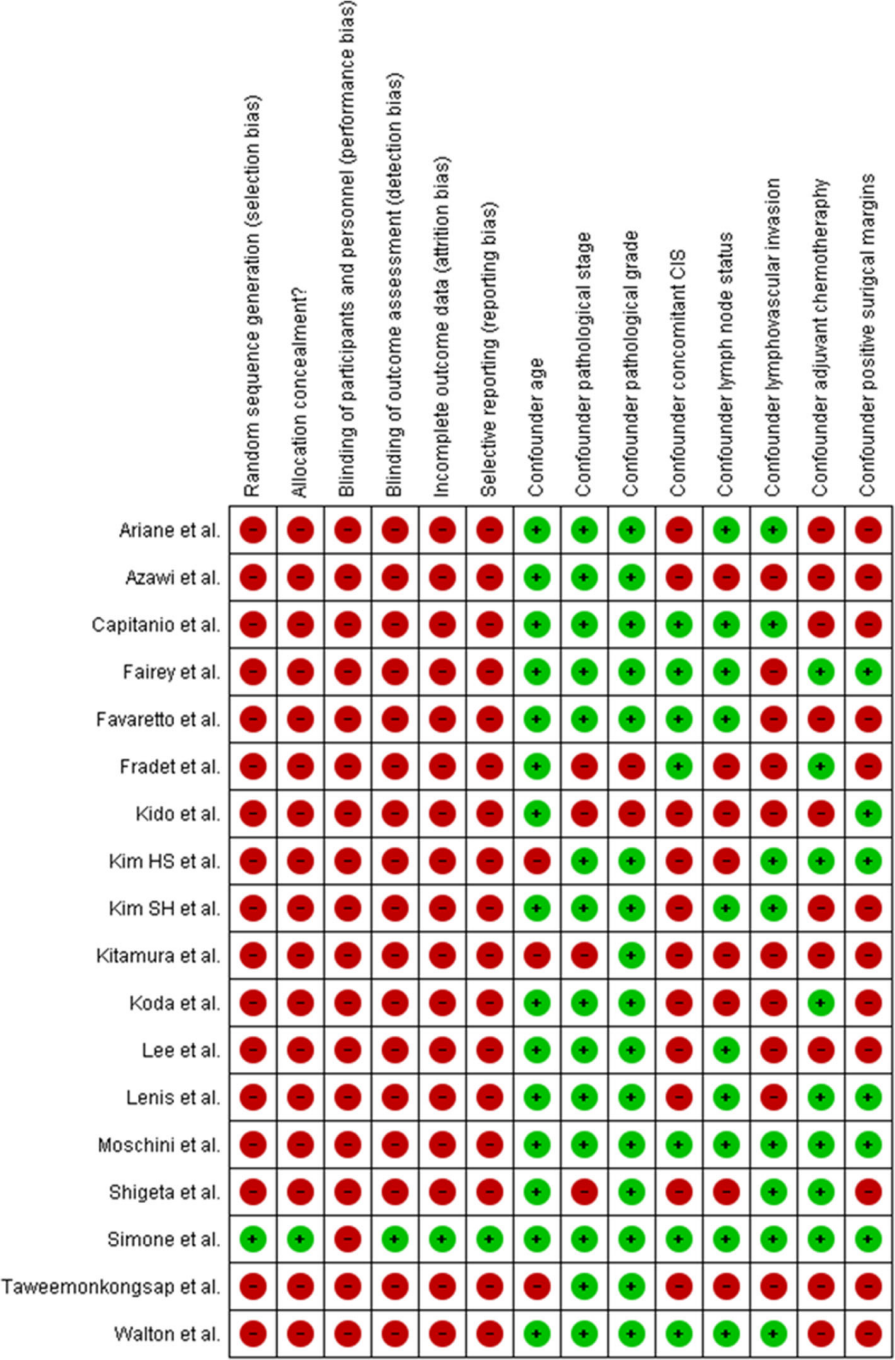
The risk of bias and confounding assessment for all included studies. Green circles represent a low risk of bias and confounding; red circles represent a high risk of bias and confounding

All studies, with the exception of the RCT, carried a high RoB, which was primarily related to their retrospective design. Assessment of RoB and confounding for each individual study was presented in Fig. 2. Overall RoB and confounding was additionally provided in Supplementary Figure 1. In 8 of 17 retrospective studies, multivariate analyses were adjusted for the effect of at least five confounders, with age, pathological stage, and grade being the most common [11, 13, 14, 18, 19, 23, 24, 27]. Matching techniques (propensity-score matching or inverse probability weighting) were implemented in four of them [17, 19, 24, 25].

### Meta-analysis results

For each outcome of interest (CSS, OS, IVRFS, RFS), we performed main analyses comprising data from main cohorts of all available publications. Subsequently, we conducted subgroup analyses of pT3/T4 and pTany N+ populations. Surgical access (transperitoneal or retroperitoneal) and study design (retrospective or RCT) were used as stratification variables in additional subgroup analyses.

**Fig. 3:**
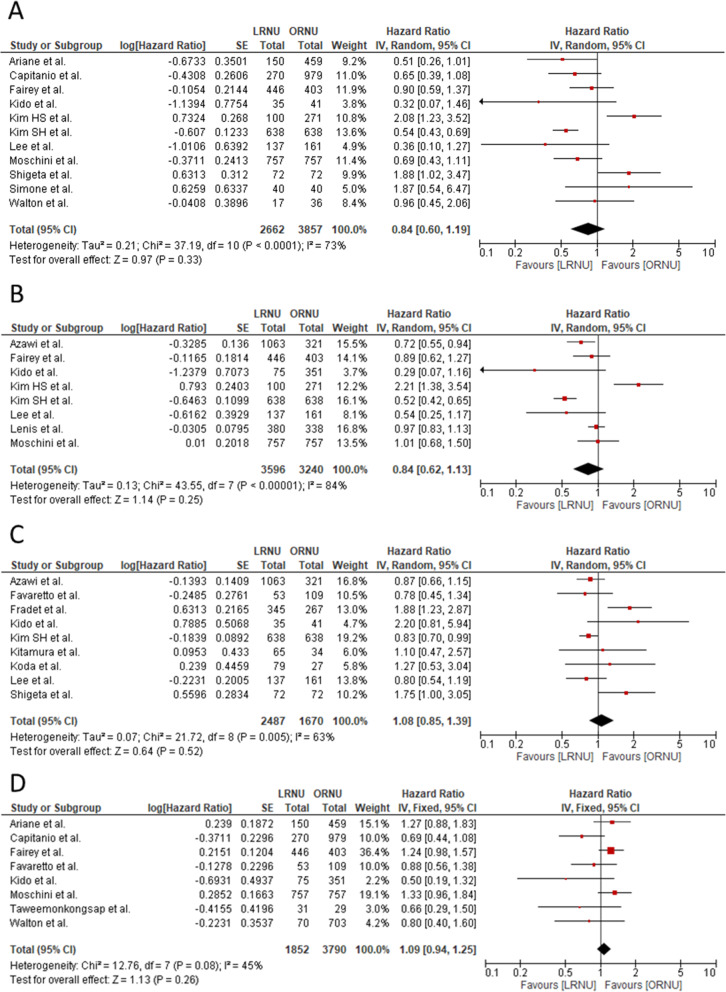
Forest plot comparing survival parameters in patients undergoing LRNU vs. ORNU. **a** Cancer-specific survival. **b** Overall survival. **c** Intravesical recurrence-free survival. **d** Recurrence-free survival. CI, confidence interval; IV, inverse variance; LRNU, laparoscopic radical nephroureterectomy; ORNU, open radical nephroureterectomy; SE, standard error

CSS data were reported in 11 included articles [5, 11, 13, 14, 17–19, 22, 24, 25, 27]. Significant heterogeneity was observed among the studies (*I*^2^ = 73%; *p* < 0.001); therefore, a random-effects model was used to analyze the outcome. A forest plot of HR and 95% CI for CSS is presented in Fig. 3a. The results of pooled analysis revealed no significant difference in CSS between LRNU and ORNU (HR 0.84, 95% CI 0.60–1.19, *p* = 0.33). The results of asymmetry tests did not show any evidence of publication bias (Supplementary Table 1; funnel plot was shown in Supplementary Figure 2A).

OS data were reported in eight included articles [12, 14, 17–19, 22–24]. Significant heterogeneity was observed among the studies (*I*^2^ = 84%; *p* < 0.001); therefore, a random-effects model was used to analyze the outcome. A forest plot of HR and 95% CI for OS is presented in Fig. 3b. The results of pooled analysis revealed no significant difference in OS between LRNU and ORNU (HR 0.84, 95% CI 0.62–1.13, *p* = 0.25). The results of asymmetry tests did not show any evidence of publication bias (Supplementary Table 1; funnel plot was shown in Supplementary Figure 2B).

IVRFS data were reported in nine included articles [12, 15–18, 20–22, 25]. Significant heterogeneity was observed among the studies (*I*^2^ = 63%; *p* = 0.005); therefore, a random-effects model was used to analyze the outcome. A forest plot of HR and 95% CI for IVRFS is presented in Fig. 3c. The results of pooled analysis revealed that LRNU and ORNU were comparable in terms of IVRFS (HR 1.08, 95% CI 0.85–1.39, *p* = 0.52). The results of asymmetry tests showed potential evidence of publication bias (Supplementary Table; funnel plot was shown in Supplementary Figure 2C).

RFS data were reported in eight articles [11, 13–15, 17, 24, 26, 27]. Significant heterogeneity was not observed among the studies (*I*^2^ = 45%; *p* = 0.08); therefore, a fixed-effects model was used to analyze the outcome. A forest plot of HR and 95% CI for RFS is presented in Fig. 3d. The results of pooled analysis revealed that LRNU and ORNU were comparable in in terms of RFS (HR 1.09, 95% CI 0.94–1.25, *p* = 0.26). The results of asymmetry tests did not show any evidence of publication bias (Supplementary Table 1; funnel plot was shown in Supplementary Figure 2D).

The results of prespecified subgroup analyses for pT3/T4 and pTany N+ cohorts did not confirm any statistically significant differences between LRNU and ORNU in terms of any oncological outcome. Also, no association between particular surgical access and improved survival parameters was found. Detailed results of all subgroup analyses were presented in Table 3. The results of meta-regression models did not show any clear source of heterogeneity between the studies (Supplementary Table 2).

**Table 3:** Subgroup analysis comparing oncological outcomes of LRNU vs. ORNU, stratified by pathological stage, surgical route, and study design

Outcome	Variable	Subgroup	No. of studies [reference]	No. of patients LRNU/ORNU	HR [95% CI] LRNU vs. ORNU	***P*** value	Heterogeneity (%)
**CSS**	Pathological stage	pT3/T4	5 [5, 18, 19, 25, 27]	420/725	1.51 [0.95–2.38]	0.08	74
		pTany N+	2 [19, 27]	33/77	0.79 [0.43–1.45]	0.44	32
	Surgical access (LRNU)	Transperitoneal	6 [5, 11, 18, 19, 22, 27]	1082/1605	0.77 [0.41–1.44]	0.41	83
		Retroperitoneal	1 [17]	35/41	0.32 [0.07–1.46]	0.14	NA
	Study design^#^	Retrospective	10 [11, 13, 14, 17–19, 22, 24, 25, 27]	2622/3817	0.81 [0.57–1.15]	0.24	74
		Randomized controlled trial	1 [5]	40/40	1.87 [0.54–6.47]	0.26	NA
**OS**	Pathological stage	pT3/T4	2 [19, 23]	285/377	1.45 [0.50–4.23]	0.50	91
		pTany N+	2 [18, 19]	115/145	0.92 [0.53–1.59]	0.76	58
	Surgical access (LRNU)	Transperitoneal	3 [18, 19, 22]	875/1070	0.86 [0.32–2.35]	0.77	93
		Retroperitoneal	1 [17]	75/351	0.29 [0.07–1.16]	0.08	NA
**IVRFS**	Pathological stage	pT3/T4	2 [19, 25]	323/337	1.14 [0.53–2.46]	0.74	85
		pTany N+	1 [19]	31/29	1.48 [0.68–3.22]	0.32	NA
	Surgical access (LRNU)	Transperitoneal	2 [19, 22]	775/799	0.83 [0.70–1.07]	0.06	0
		Retroperitoneal	2 [17, 21]	114/68	1.61 [0.84–3.11]	0.15	0
**RFS**	Pathological stage	pT3/T4	2 [14, 27]	143/347	1.00 [0.66–1.51]	0.98	0
		pTany N+	2 [14, 27]	20/90	1.59 [0.62–4.07]	0.33	30
	Surgical access (LRNU)	Transperitoneal	2 [11, 27]	220/1162	1.15 [0.83–1.59]	0.40	25
		Retroperitoneal	2 [17, 26]	106/380	0.59 [0.31–1.10]	0.10	0

^#^Subgroup analysis was possible only for CSS

*Abbreviations*: CI, confidence interval; CSS, cancer-specific survival; HR, hazard ratio; IVRFS, intravesical recurrence-free survival; LRNU, laparoscopic radical nephroureterectomy; NA, not applicable; ORNU, open radical nephroureterectomy; OS, overall survival; RFS, recurrence-free survival

## Discussion

In the present meta-analysis, we attempted to provide cumulatively summarized evidence regarding oncological outcomes of LRNU compared with those of ORNU. Our analyses demonstrated that laparoscopic and open approaches were equivalent in terms of oncological outcomes, including CSS, OS, IVRFS, and RFS.

The first studies comparing LRNU to ORNU in patients with UTUC were published in 1993. Subsequently, multiple authors have demonstrated that LRNU could be associated with equivalent or significantly better perioperative outcomes compared to ORNU, including parameters such as reduced blood loss, faster recovery, or shorter hospital stay [28, 29]. However, debate continues within the urologic community about which approach is associated with superior oncological outcomes and definitive conclusions remain a matter of controversy.

Many investigators have proposed hypotheses regarding the possible inferiority of LRNU compared to ORNU in terms of oncological outcomes. First, concerns about the oncologic safety of LRNU have been attributed to the pneumoperitoneal environment. It has been postulated that manipulation within a tumor mass during increased intra-abdominal pressure might increase the risk of recurrence, because of gravitational effects leading to seeding and implanting cancer cells in the bladder or retroperitoneal space, especially in locally advanced tumors [30]. Nonetheless, the constant development of the LRNU technique, including use of closed systems and endobags, has clearly reduced this risk. In our prespecified subgroup analyses for locally advanced disease, stratified for pT3/T4 and pTany N+ populations, we did not confirm any statistically significant differences between LRNU and ORNU in terms of any survival parameter. Also, no significant differences were found regarding particular surgical access (transperitoneal vs. retroperitoneal).

Second, management of bladder cuff might play a critical oncological role. Unfortunately, we could not reliably perform subgroup analyses based on different approaches, because most studies included in the present meta-analysis reported heterogeneous populations in terms of bladder cuff management. The oncologic importance of complete bladder cuff excision is underscored by the fact that the risk of tumor recurrence within this residual ureteric stump can be as high as 30–65% [31]. The assumption of worse survival outcomes in patients treated with LRNU and laparoscopic excision of the bladder cuff and distal ureter was based on the results of a sole RCT, which was also included in this meta-analysis. In this RCT, all patients underwent laparoscopic bladder cuff excision and LRNU was associated with significantly worse CSS and metastatic-free survival [5]. Recently, Shigeta et al. evaluated the oncological outcomes of pure LRNU (laparoscopic bladder cuff resection) compared with conventional LRNU (open bladder cuff resection) using a multi-institutional collaboration dataset. The 3-year IVRFS rate was significantly lower in the pure LRNU group compared to the conventional LRNU group (41.8% vs. 66.6%, *p* = 0.004). In multivariate analysis, pure LRNU was found to be an independent risk factor for worse IVRFS. Although no significant differences in 3-year RFS were found between the two methods, atypical recurrence sites (brain, sigmoid colon, vagina, peritoneum) were observed in the pure LRNU group [32]. The potential explanation for these findings might be challenging technical aspects of laparoscopic bladder cuff excision, such as closure of the bladder, which increases the risk of urine spillage in the surgical bed. The pure laparoscopic approach also risks leaving behind viable ureteral mucosa [31, 32]. However, further RCTs would be necessary to confirm the superiority of particular bladder cuff excision methods.

It has to be emphasized that administration of perioperative chemotherapy could potentially impact on the oncological outcomes of patients with UTUC treated with LRNU or ORNU. In a recent meta-analysis, Leow et al. found an OS and CSS benefit for NAC over radical nephroureterectomy (RNU) alone [33]. Furthermore, after pooling data from 29 studies, including results of the POUT trial (NCT01993979), the authors showed significant OS, CSS, and disease-free survival benefits in those who received AC compared with those who underwent RNU alone [33]. Although several papers included in our meta-analysis were adjusted for the AC confounder, only one provided data for the separated cohort of patients receiving AC, reporting no differences between LRNU and ORNU groups in terms of CSS and RFS [27]. Moreover, it has to be emphasized that in the majority of selected articles, patients receiving NAC were initially excluded or such data were not reported. As the RNU alone fails to manage significant number of high-risk UTUCs, there is an urgent need to combine surgery with systemic cancer control strategies. It was demonstrated by Margulis et al. that administration of four NAC cycles of accelerated methotrexate, vinblastine, doxorubicin, and cisplatin allows to achieve 14% complete pathological response rate (ypT0N0) in patients with high grade UTUC. Further, final pathological stage ypT1 or less was reported in more than 60% of patients [34]. Despite small sample size, the results of this study support the NAC feasibility in patients with UTUC, who are initially qualified for nephroureterectomy. Notwithstanding, current evidences are still lacking and future prospective trials are necessary to make definitive conclusions.

Several systematic reviews and meta-analyses that assessed the oncological outcomes of LRNU versus ORNU have been published to date. Initially, Ni et al. showed significantly higher rates of 5-year CSS for patients who underwent LRNU compared to those who underwent ORNU (9%, *p* = 0.03). Conversely, the overall recurrence rate and bladder recurrence rate were significantly lower, at 15% (*p* = 0.01) and 17% (*p* = 0.02), respectively. No statistically significant differences in other survival parameters (2-year CSS, 5-year RFS, 5-year OS, 2-year OS, and metastasis rates) were found between LRNU and ORNU. The interpretation of these data was limited because patients managed with a laparoscopic approach were more likely to have Ta/Tis or T1 disease and less likely to have T3 or T4 lesions [35]. Subsequently, Zhang et al. in their meta-analysis showed no differences in the OS, IVRFS, and unspecified RFS between LRNU and ORNU. However, improvements in the extravesical RFS and CSS were observed in the LRNU group. The results of this study should be interpreted cautiously, because of multiple methodological flaws, such as mixing the time-to-event data (HR) with the odds ratio [36]. Furthermore, Peyronnet et al. in their systematic review published in 2018 comprehensively reviewed the available evidence and suggested that the oncological outcomes of LRNU may be poorer than those of ORNU in patients with locally advanced high-risk (pT3/pT4 high-grade) UTUC, which was not confirmed in our meta-analysis [37]. In the most recent meta-analysis conducted by Liu et al., no significant differences in the rates of both 2-year and 5-year RFS, CSS, and OS were observed; however, no detailed subgroup analyses were performed [6].

All of the aforementioned meta-analytic studies have implicated potential risk factors and selection biases resulting from inclusion of retrospective studies. Through inclusion of data from multivariable analyses only (mainly adjusted for the effects of major confounders), we could minimize bias and establish the highest level of comparability to date. Since the publication of the most recent meta-analysis [6], seven new articles including overall 5760 participants have been published [12, 17, 19, 22–25]. Although all novel publications had retrospective design, matching techniques (propensity score matching, inverse probability weighting) were implemented in majority of them (contrary to the previously published original articles), making their results more reliable and less biased by retrospective design. Thus, the quality of available data improved since the publication of the last meta-analysis. Also, the major novelty of the current study are the subgroup analyses on non-organ confined patient.

Despite several strengths, this study is not devoid of limitations. First, the strength of the conclusions that can be drawn from our meta-analysis is still limited by the fact that almost all included studies were retrospective, with their own unavoidable limitations. Second, long-term follow-up was not reported in several studies. Third, the adjustments for confounders in the Cox regression analyses were not uniform in the included trials, which might introduce additional bias. Fourth, additional data regarding surgical approach (e.g., distal ureter management, bladder cuff excision, extent of LN dissection) were not uniformly reported and the influence of such significant heterogeneity could not be fully excluded. Fifth, the selection of the surgical procedure in patients with UTUC depends primarily on clinical stage; however, our subgroup analyses stratified by tumor stage were performed according to pathological stage, which potentially limits the conclusions with respect to current daily practice. Sixth, the results of subgroup analyses should be interpreted carefully, as available data are still limited.

## Conclusions

The present meta-analysis of the current evidences confirms that LRNU and ORNU have comparable oncological outcomes in patients with UTUC, even in those with locally advanced disease. Further multicenter RCTs with large sample sizes and uniform data regarding specific surgical procedures, such as bladder cuff excision, are required to establish definitive conclusions.

## Supplementary Information

**Additional file 1: Supplementary Figure 1.** Overall risk of bias graph.

**Additional file 2: Supplementary Figure 2.** Funnel plot for the evaluation of potential publication bias: (A) cancer-specific survival; (B) overall survival; (C) intravesical recurrence-free survival; (D) recurrence-free survival.

**Additional file 3: Supplementary Table 1.** Results of asymmetry tests for publication bias assessment.

**Additional file 4: Supplementary Table 2.** Meta-regression according to methodological covariates.

## Data Availability

All data generated or analyzed during this study are included in this published article.

## Abbreviations

AC: Adjuvant chemotherapy
CI: Confidence interval
CIS: Carcinoma in situ
CSS: Cancer-specific survival
HR: Hazard ratio
IVRFS: Intravesical recurrence-free survival
LN: Lymph nodes
LRNU: Laparoscopic radical nephroureterectomy
LVI: Lymphovascular invasion
NAC: Neoadjuvant chemotherapy
NOS: Newcastle-Ottawa Scale
OS: Overall survival
ORNU: Open radical nephroureterectomy
PSM: Positive surgical margins
R: Retrospective
RCT: Randomized controlled trial
RFS: Recurrence-free survival
RoB: Risk of bias
UTUC: Upper tract urothelial carcinoma
