# Supplementary figures and images for: Oncological outcomes of laparoscopic versus open nephroureterectomy for the treatment of upper tract urothelial carcinoma: an updated meta-analysis
Source: World J Surg Oncol. 2021 Apr 21;19:129. doi: 10.1186/s12957-021-02236-z (PMC8061074; doi:10.1186/s12957-021-02236-z)

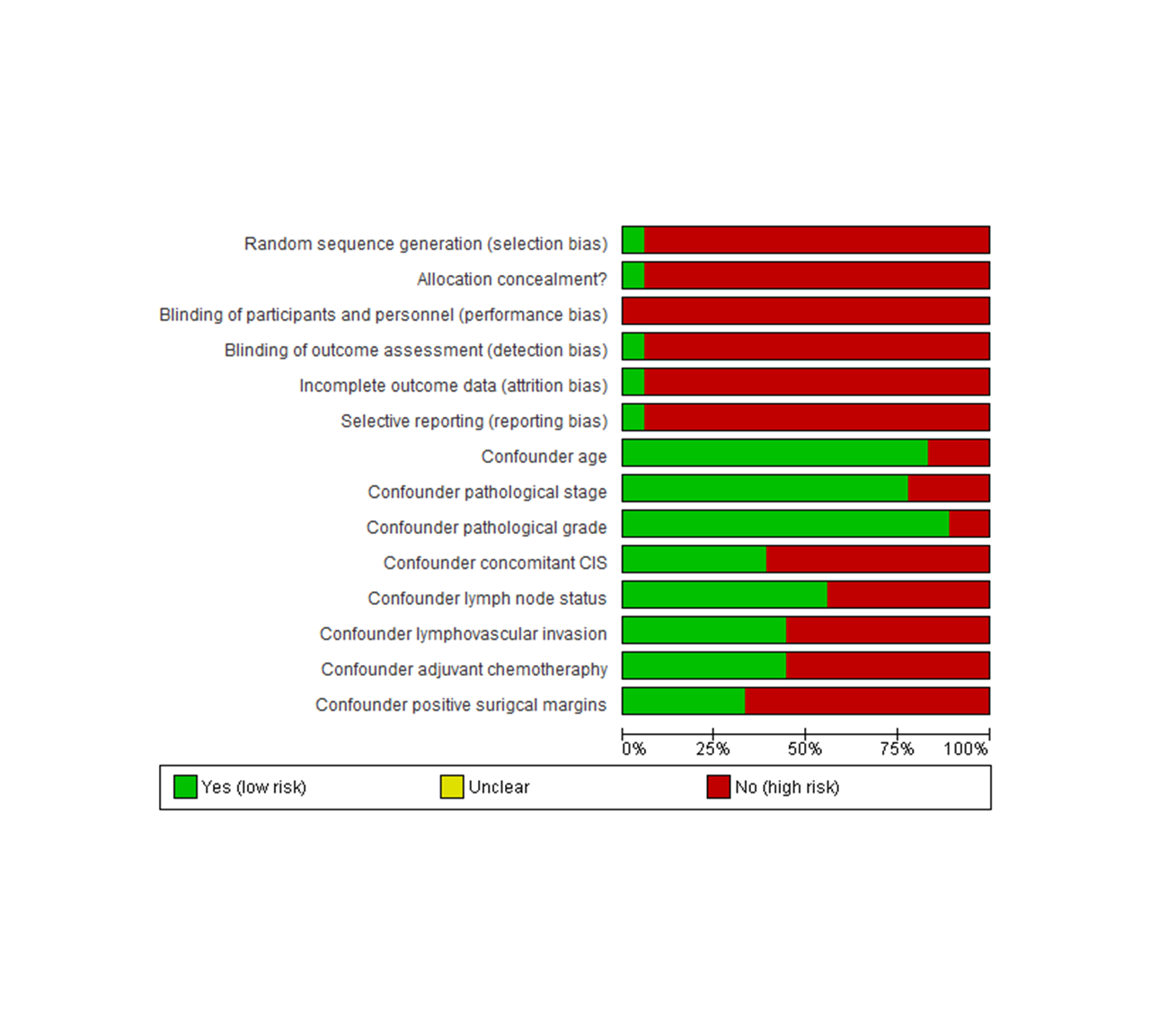

Supplement: Supplementary file 1 — Additional file 1: Supplementary Figure 1. Overall risk of bias graph. [file 12957_2021_2236_MOESM1_ESM.tif]

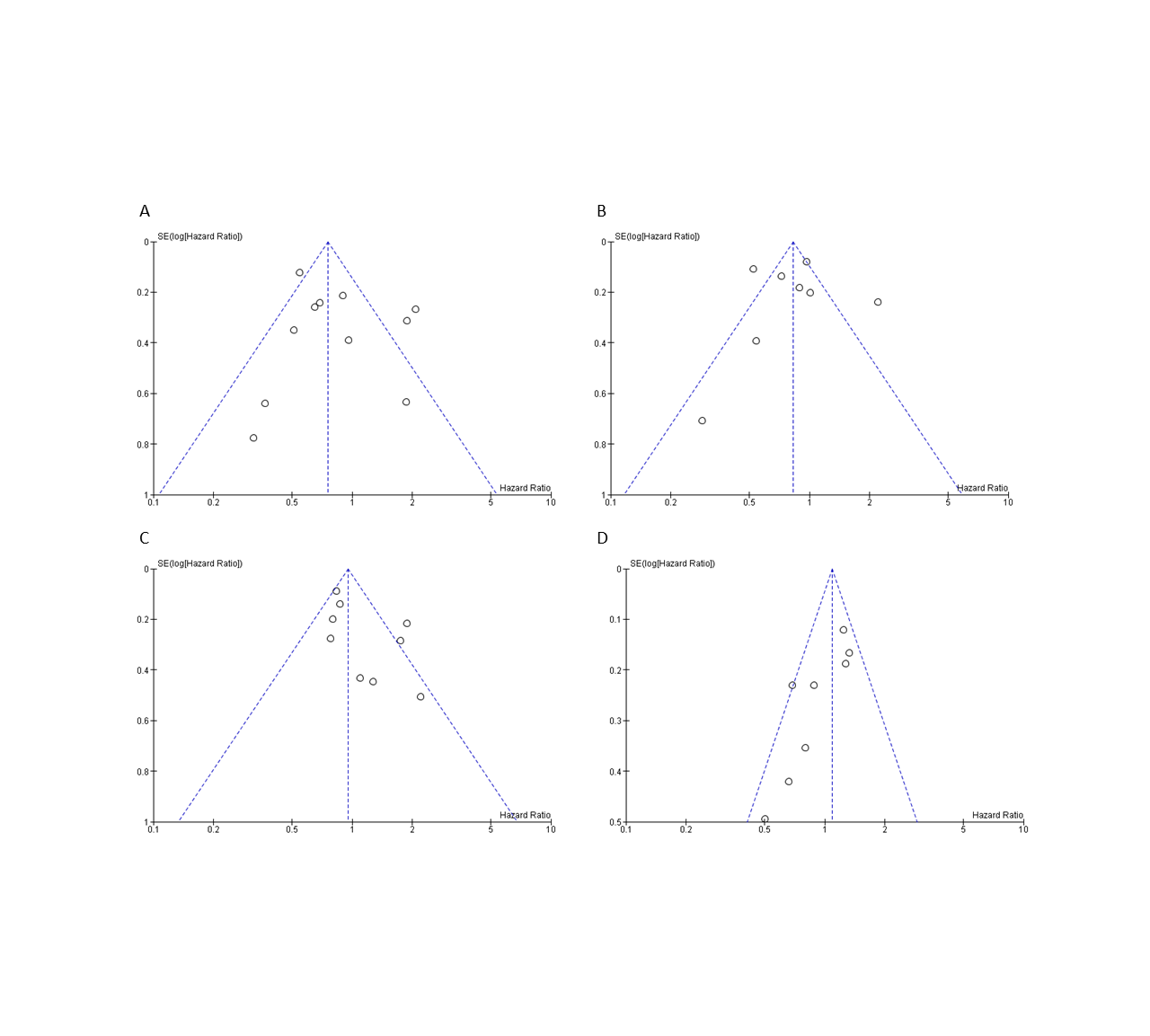

Supplement: Supplementary file 2 — Additional file 2: Supplementary Figure 2. Funnel plot for the evaluation of potential publication bias: (A) cancer-specific survival; (B) overall survival; (C) intravesical recurrence-free survival; (D) recurrence-free survival. [file 12957_2021_2236_MOESM2_ESM.tif]
