# Supplementary material for: Oncological outcomes of laparoscopic versus open nephroureterectomy for the treatment of upper tract urothelial carcinoma: an updated meta-analysis
Source: World J Surg Oncol. 2021 Apr 21;19:129. doi: 10.1186/s12957-021-02236-z (PMC8061074; doi:10.1186/s12957-021-02236-z)
Supplement: Supplementary file 3 — Additional file 3: Supplementary Table 1. Results of asymmetry tests for publication bias assessment. [file 12957_2021_2236_MOESM3_ESM.docx]

**Supplementary Table 1.** Results of asymmetry tests for publication bias assessment

| **Outcome** | **Egger’s test**  (p-value) | **Begg-Mazdumar’s test**  (p-value) |
| --- | --- | --- |
| Cancer-specific survival | 0.49 | 0.67 |
| Overall survival | 0.85 | 0.17 |
| Intravesical recurrence-free survival | 0.01* | 0.01* |
| Recurrence free survival | 0.08 | 0.13 |

* Statistically significant difference
