# Supplementary material for: Oncological outcomes of laparoscopic versus open nephroureterectomy for the treatment of upper tract urothelial carcinoma: an updated meta-analysis
Source: World J Surg Oncol. 2021 Apr 21;19:129. doi: 10.1186/s12957-021-02236-z (PMC8061074; doi:10.1186/s12957-021-02236-z)
Supplement: Supplementary file 4 — Additional file 4: Supplementary Table 2. Meta-regression according to methodological covariates. [file 12957_2021_2236_MOESM4_ESM.docx]

**Supplementary Table 2.** Meta-regression according to methodological covariates

| **Outcome** | **Variable** | **Number of estimates** | **P-value in univariable analysis** | **Multivariable adjusted R^2^ of the model** (%) | **Multivariable adjusted R^2^** (%) |
| --- | --- | --- | --- | --- | --- |
| **Cancer-specific survival**  (CSS) | **Site** |  | 0.76 | 55.5% | 16.2% |
|  | Multicentre | 8 |  |  |  |
|  | Single-centre | 3 |  |  |  |
|  | **Sample size** |  | 0.54 |  | 12.9% |
|  | < 500 | 5 |  |  |  |
|  | ≥ 500 | 6 |  |  |  |
|  | **Geographic location** |  | 0.86 |  | 13% |
|  | Americas | 1 |  |  |  |
|  | Europa | 2 |  |  |  |
|  | Asia | 5 |  |  |  |
|  | International | 3 |  |  |  |
|  | **Surgical access (LRNU)** |  | 0.92 |  | 13.4% |
|  | Transperitoneal | 6 |  |  |  |
|  | Retroperitoneal | 1 |  |  |  |
|  | Both/not reported | 4 |  |  |  |
| **Overall survival**  (OS) | **Site** |  | 0.32 | 61.2% | 15.7% |
|  | Multicentre | 6 |  |  |  |
|  | Single-centre | 2 |  |  |  |
|  | **Sample size** |  | 0.46 |  | 16% |
|  | < 500 | 4 |  |  |  |
|  | ≥ 500 | 4 |  |  |  |
|  | **Geographic location** |  | 0.56 |  | 17.2% |
|  | Americas | 2 |  |  |  |
|  | Europa | 1 |  |  |  |
|  | Asia | 4 |  |  |  |
|  | International | 1 |  |  |  |
|  | **Surgical access (LRNU)** |  | 0.88 |  | 12.3% |
|  | Transperitoneal | 3 |  |  |  |
|  | Retroperitoneal | 1 |  |  |  |
|  | Both/not reported | 4 |  |  |  |
| **Intravesical recurrence-free survival**  (IVRFS) | **Site** |  | 0.73 | 56% | 17.6% |
|  | Multicentre | 6 |  |  |  |
|  | Single-centre | 3 |  |  |  |
|  | **Sample size** |  | 0.96 |  | 7.3%% |
|  | < 500 | 6 |  |  |  |
|  | ≥ 500 | 3 |  |  |  |
|  | **Geographic location** |  | 0.47 |  | 20.5% |
|  | Americas | 2 |  |  |  |
|  | Europa | 1 |  |  |  |
|  | Asia | 6 |  |  |  |
|  | International | - |  |  |  |
|  | **Surgical access (LRNU)** |  | 0.45 |  | 10.6% |
|  | Transperitoneal | 2 |  |  |  |
|  | Retroperitoneal | 2 |  |  |  |
|  | Both/not reported | 5 |  |  |  |
| **Recurrence-free survival** (RFS) | **Site** |  | 0.87 | 67.6% | 15.9% |
|  | Multicentre | 6 |  |  |  |
|  | Single-centre | 2 |  |  |  |
|  | **Sample size** |  | 0.67 |  | 10.1% |
|  | < 500 | 3 |  |  |  |
|  | ≥ 500 | 5 |  |  |  |
|  | **Geographic location** |  | 0.42 |  | 22.3% |
|  | Americas | 2 |  |  |  |
|  | Europa | 1 |  |  |  |
|  | Asia | 2 |  |  |  |
|  | International | 3 |  |  |  |
|  | **Surgical access (LRNU)** |  | 0.91 |  | 19.3% |
|  | Transperitoneal | 2 |  |  |  |
|  | Retroperitoneal | 2 |  |  |  |
|  | Both/not reported | 4 |  |  |  |

**Abbreviations: LRNU:** laparoscopic radical nephroureterectomy
